# The COP9 signalosome complex regulates fungal development and virulence in the wheat scab fungus *Fusarium graminearum*

**DOI:** 10.3389/fmicb.2023.1179676

**Published:** 2023-04-24

**Authors:** Ahai Chen, Yiyi Ren, Xingmin Han, Chao Liu, Yifan Zhou, Chenghui Xu, Hao Qi, Zhonghua Ma, Yun Chen

**Affiliations:** State Key Laboratory of Rice Biology, The Key Laboratory of Molecular Biology of Crop Pathogens and Insects, Institute of Biotechnology, Zhejiang University, Hangzhou, China

**Keywords:** the COP9 signalosome complex, deoxynivalenol (DON), fungal development, virulence, *Fusarium graminearum*

## Abstract

The COP9 signalosome (Csn) complex is an evolutionarily conserved complex that regulates various important cellular processes. However, the function of the Csn complex in pathogenic fungi remains elusive. Here, the distribution of Csn subunits in the fungal kingdom was surveyed, and their biological functions were systematically characterized in the fungal pathogen *Fusarium graminearum*, which is among the top 10 plant fungal pathogens. The results obtained from bioinformatic analyses suggested that the *F. graminearum* Csn complex consisted of seven subunits (Csn1–Csn7) and that Csn5 was the most conserved subunit across the fungi kingdom. Yeast two-hybrid assays demonstrated that the seven Csn subunits formed a complex in *F. graminearum.* The Csn complex was localized to both the nucleus and cytoplasm and necessary for hyphal growth, asexual and sexual development and stress response. Transcriptome profiling revealed that the Csn complex regulated the transcription abundance of *TRI* genes necessary for mycotoxin deoxynivalenol (DON) biosynthesis, subsequently regulating DON production to control fungal virulence. Collectively, the roles of the Csn complex in *F. graminearum* were comprehensively analyzed, providing new insights into the functions of the Csn complex in fungal virulence and suggesting that the complex may be a potential target for combating fungal diseases.

## 1. Introduction

The COP9 signalosome (Csn) complex is an evolutionarily conserved protein complex that was originally identified in *Arabidopsis thaliana* from a genetic screen for mutants with the COP/DET/FUS (constitutively photomorphogenic/de-etiolated/fusca) phenotype. Under darkness, Csn-deficient plants exhibit developmental patterns that are normally visible in the light (Wei and Deng, 1992; Wei et al., 1994). A canonical Csn complex is composed of eight subunits (Csn1–8); Csn5 and Csn6 harbor an MPN (Mov34 and Pad1p N-terminal) domain, while the remaining six subunits contain a PCI (proteasome, COP9, and initiation Factor 3) domain (Wei et al., 2008; Qin et al., 2020). Metalloprotease Csn5 contains a conserved JAMM (JAB1 MPN structural domain met isozyme) motif that is activated by zinc ions. This motif is not present in Csn6; therefore, Csn6 is classified as an MPN subunit, while Csn5 is classified as MPN+ (Cope et al., 2002; Verma et al., 2002). A complete eight-subunit Csn complex consisting of two MPN and six PCI-domain-containing proteins has been identified in higher organisms, including humans, files, mice and plants (Wei et al., 1998; Oron et al., 2002; Hetfeld et al., 2005; Rubio et al., 2005). However, the composition of the Csn complex varies between species in fungi. Eight subunits of the Csn complex are present in *Aspergillus nidulans* (Busch et al., 2007), while a smaller version of the Csn complex is present in *Neurospora crassa*, which lacks Csn8 (He et al., 2005; Wang et al., 2010). Csn6 and Csn8 are not present in the fission yeast *Schizosaccharomyces pombe*, while Csn4, Csn6 and Csn8 are missing from the budding yeast *Saccharomyces cerevisiae* (Liu et al., 2003; Maytal-Kivity et al., 2003), indicating that fungi are a good model system for studying Csn conservation and evolution.

One of the major functions of the Csn complex is to regulate the activity of cullin-RING ligase (CRL) families (Wei et al., 2008; Qin et al., 2020). CRLs are the major group of ubiquitin E3 complexes and always share a common cullin/RING-E2 module (Petroski and Deshaies, 2005; Bosu and Kipreos, 2008; Hotton and Callis, 2008). CRLs are activated when the ubiquitin-like protein Nedd8 (neural precursor cell expressed, developmentally downregulated 8) is covalently linked to the cullin proteins of CRLs (Wang et al., 2010). Conversely, the COP9 signalosome complex is required for promoting the removal of Nedd8 from cullins, subsequently negatively regulating the E3 ubiquitin ligase activity of CRLs (Lyapina et al., 2001), which is dependent on the JAMM motif of the Csn5 subunit (Lyapina et al., 2001; Schwechheimer et al., 2001; Cope et al., 2002). Neddylated and active E3 ubiquitin ligases are crucial for labeling proteins for the proteasome and subsequent protein degradation (Petroski and Deshaies, 2005). Therefore, the Csn complex regulates ubiquitin-dependent protein degradation by mediating the neddylation status and activity of E3 ubiquitin ligases. In addition to the regulation of protein degradation, the Csn complex regulates transcription (Chamovitz, 2009). For example, the Csn2 Alien isoform has been shown to act on chromatin directly, regulating nucleosome assembly protein Nap1-mediated nucleosome assembly and thus participating in the regulation of gene expression (Eckey et al., 2007). The Csn complex mediates multiple cellular and developmental processes in plants, mammals and fungi due to the critical roles of the Csn complex in regulating gene expression and the activity of CRLs and subsequent protein degradation (Wei et al., 2008; Kato and Yoneda-Kato, 2009; Braus et al., 2010). In addition to its typical role in light signaling in *Arabidopsis*, the Csn complex plays critical roles in hormone signaling, temperature signaling, and cell cycle progression (Schwechheimer and Isono, 2010; Qin et al., 2020). In mammals, the Csn complex is associated with signal transduction, cycle regulation and autophagy (Kato and Yoneda-Kato, 2009; Lee et al., 2011; Su et al., 2013). More importantly, several human diseases, such as preeclampsia, cardiovascular diseases, and cancer, are associated with dysfunctional Csn complex (Richardson and Zundel, 2005; Lee et al., 2011; Cayli et al., 2012; Milic et al., 2019). Given the biomedical importance of this complex, the activity of the Csn complex could be potential targets for drug development (Lim et al., 2016; Du et al., 2022). The Csn complex is not essential for the viability of fungi, unlike plants and animals (Wei et al., 2008; Braus et al., 2010; Zhao et al., 2011). The Csn in *N. crassa* is involved in asexual development and the circadian clock (Wang et al., 2010), while the *A. nidulans* Csn plays a role in fruit body formation, secondary metabolism and light response (Busch et al., 2003, 2007; Nahlik et al., 2010). In *S. cerevisiae*, the Csn complex is important for mating pheromone response, metal uptake and lipid metabolism (Maytal-Kivity et al., 2003; Licursi et al., 2014), while the *S. pombe* Csn has a role in DNA damage and the coordination of S phase (Mundt et al., 1999, 2002). Although the biological roles of the Csn complex have been elucidated in several fungal species, the biological functions of the Csn complex remain limited, especially in fungal virulence.

Fusarium head blight (FHB), which is mainly caused by *Fusarium graminearum*, destroys small-grain cereal crops worldwide (Dean et al., 2012; Chen et al., 2022). For example, approximately 20% of the wheat area planted (over 4.5 million hectares) in China was affected by FHB, which led to over 3.41 million tons of yield loss each year between 2000 and 2018 (Chen et al., 2019). In addition to causing yield losses, mycotoxins such as zearalenone and deoxynivalenol (DON) produced by *F. graminearum* are hazardous to human and livestock health (Desjardins, 2003; Pestka and Smolinski, 2005). However, managing FHB remains a challenge due to limited FHB-resistant cultivars and prevalent fungicide resistance. Recently, it has been reported that a host-induced gene silencing (HIGS) strategy that targets fungal virulence factors significantly enhanced wheat resistance against FHB (Cheng et al., 2015; Wang et al., 2020). Thus, clarifying the molecular mechanisms that underly *F. graminearum* virulence will provide new potential targets for developing HIGS-based FHB-resistant wheat cultivars and antifungal agents.

In this study, the homolog of the Csn complex was identified in *F. graminearum*, and the interactions among the Csn subunits were clarified. Moreover, our findings revealed that the Csn complex localized to the nucleus and cytoplasm and was important for fungal development and virulence of *F. graminearum*. Furthermore, the regulatory mechanisms of the Csn complex in fungal development and virulence were further explored and we found that the complex regulated ribosome assembly, protein synthesis and nutrition metabolism, which are associated with fungal growth and development. In addition, the Csn complex is necessary for the normal stress response, toxisome formation and DON production, which are crucial for *F. graminearum* virulence. Based on these results, we conclude that the Csn complex is conserved and plays a crucial role in fungal development and virulence in *F. graminearum*, providing novel possibilities and targets to control fungal diseases.

## 2. Materials and methods

### 2.1. Identification of the COP9 signalosome subunits in 1,642 fungal species

To identify the Csn subunits in fungi, BLAST 2.12.0+ was used to build a local library for 1,642 fungal genomes. The Csn subunits on 1,642 fungal genomes were searched using the protein sequences of 8 Csn subunits from *A. nidulans* as references (Busch et al., 2007; Li et al., 2021) and 8,630 candidate target sequences were identified in this study. The number and average similarity of 8 Csn subunits were counted at the phylum and subphylum level.

### 2.2. Strains and cultural conditions

PH-1 (NRRL 31084), the wild-type strain of *F. graminearum*, was used as the host strain to generate gene deletion mutants for Csn subunits. The obtained mutants were stored and cultured on PDA (potato dextrose agar) medium. PDA medium and carboxymethyl cellulose (CMC) liquid medium were used for the analysis of vegetative growth and conidiation assay, respectively (Chen et al., 2020). Carrot medium (20 carrot and 2% agar) and trichothecene biosynthesis induction (TBI) medium were used for the analysis of perithecium formation and DON production, respectively (Tang et al., 2018). For stress response, the tested strains were cultured on a complete medium (CM) supplemented with different stress agents and the growth inhibition rates were measured after incubation at 25°C for 3 days. To detect the localization of the Csn complex, the mycelial plugs (5 mm) of the strain expressing of GFP-Csn5 and the nuclear marker histone 1 (H1)-mCherry were cultivated in liquid complete medium at 25°C for 16 h in a shaker (180 rpm) and the localization of Csn5 was examined under confocal microscopy. Similar growth conditions were used to detect the localizations of other Csn subunits.

### 2.3. Generation of mutants, complementation strains, and tag-fusion cassettes

To generate gene deletion constructs for Csn subunits, 500–1,200 bp upstream and downstream flanking sequences of targeted genes were amplified and were further fused with the hygromycin phosphotransferase (*HPH*) selectable maker using the double-joint PCR as previously described (Yu et al., 2004). The obtained constructs were transformed into the protoplasts of the host strain following protocols described previously (Hou et al., 2002). The PCR assay was performed to screen positive gene knockout mutants. To generate the corresponding complementation strain of mutants, open reading frame (ORF) of target gene and its native promoter were amplified, respectively and fused with GFP tag, the resulting amplicons, along with the geneticin-resistance gene were transformed in to the protoplasts of corresponding mutant. Similar strategies were conducted to generate other strains expressing Flag-fusion constructs. The primers used for genetic manipulation were presented in Supplementary Table 1.

### 2.4. Yeast two-hybrid (Y2H) assay

The Y2H assay was performed as described previously (Chen et al., 2020). Briefly, ClonExpress One Step Cloning Kit (Vazyme, Nanjing, China) was used to generate bait and prey constructs. After sequencing, the resulting constructs were transformed into *S. cerevisiae* strain AH109 following the previous protocol (Schiestl and Gietz, 1989). After transformation, the growth of the transformant was determined on synthetic medium (SD)-Leu/Trp and SD-Trp-Leu-His-Ade medium, respectively. The BD-Lam/AD-T pair and BD-53/AD-T pair were used as the negative and positive interaction control, respectively.

### 2.5. Pathogenicity and DON biosynthesis assays

The pathogenicity of tested strains was determined as described previously (Chen et al., 2020). Briefly, flowering wheat heads were inoculated with tested strains and the symptoms were recorded 14 days post-inoculation (dpi). 7-day-old wheat seeding leaves and corn silks inoculated with tested strains were cultured at 25°C and the symptoms were measured at 7 dpi. An agar plug without fungal mycelia was used as the negative control. DON detection kit (Wisestep Biotech Co., Ltd., Shenzhen, China) was used to detect the DON production of tested strains after incubation in liquid TBI medium in the dark for 7 days. To determine the transcriptional expression of the *TRI* genes, the total RNAs of tested strains were isolated by Trizol. After reverse transcription using the HiScript II 1st Strand cDNA Synthesis Kit (R212-02, Vazyme, China), quantitative real-time PCR (qRT-PCR) was performed with ChamQ SYBR qPCR Master Mix (Q311-02, Vazyme, China). Actin was used as a reference gene. The primers used in the qRT-PCR assay were presented in Supplementary Table 1.

### 2.6. Co-immunoprecipitation (Co-IP) and western blotting assays

Co-immunoprecipitation assay was conducted as previously described (Chen et al., 2020). Briefly, the strains bearing GFP-Csn5 and Flag-Csn1 or a single construct were generated in this study. The GFP-Csn5 were immunoprecipitated from total proteins of tested strains using anti-GFP agarose beads (ChromoTek, Martinsried, Germany) and Flag-Csn1 was detected from proteins co-purified with GFP-Csn5 to verify the association between Csn5 and Csn1. A similar strategy was used to detect the other associations. Western blotting assay was conducted as previously described (Chen et al., 2020). An anti-GFP (Ab32146, Abcam, Cambridge, UK) antibody and anti-Flag (A9044, Sigma, St. Louis, MO, USA) antibody were used to detect GFP-fused and Flag-fused proteins, respectively. As a reference, the tested samples were detected with the anti-GAPDH antibody (EM1101, HuaAn, Shanghai, China).

### 2.7. RNA sequencing and transcriptome analysis

There were three biological replicates for each treatment. For RNA-seq data, library construction and sequencing were performed on an Illumina HiSeq2000 (pair ends). Raw RNA-seq reads were removed of low-quality reads and adapter sequences using Trimmomatic v0.39 (Bolger et al., 2014) with default parameters. Clean reads were mapped to the reference genome using STAR v2.7.6a (Dobin et al., 2013), respectively. The read number for each gene was counted by featureCounts v1.6.0 (Liao et al., 2014) and the resulting transcript count tables were subjected for differential expression analysis using R packages edgeR v3.360 (Robinson et al., 2010) and limma v3.50.0 (Ritchie et al., 2015). Transcripts with an adjusted *P*-value of ≤ 0.05 and log_2_ fold change of ≥1 or ≤–1 were determined as differentially expressed genes. Gene Ontology (GO) enrichment analysis of differentially up- or down-expressed genes was conducted using ShinyGO v0.75 (Ge et al., 2020).

## 3. Results

### 3.1. Distribution and conservation of the Csn subunits across the fungal kingdom

**FIGURE 1:**
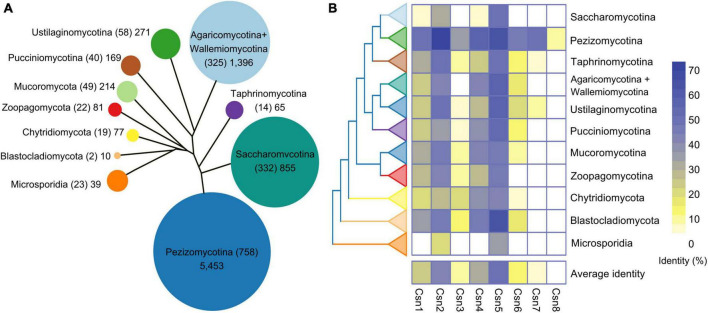
Genome-wide identification and conservation analysis of the COP9 signalosome (Csn) complex in fungi. **(A)** Number of Csn subunits at the subphylum or phylum level. The evolutionary tree shows the relationships among the various fungal groups. The area of a circle is proportional to the total amount of the Csn subunits identified in each group. The number of species is indicated in parentheses. **(B)** Conservation of the Csn subunits in fungi. The eight Csn subunits in *Aspergillus nidulans* were used as query sequences for analysis.

Previous studies showed that the number of Csn subunits was organism-specific in fungi (Liu et al., 2003; Maytal-Kivity et al., 2003; Busch et al., 2007; Wang et al., 2010). It would be interesting to systematically investigate the distribution and conservation of Csn subunits throughout the fungal kingdom. To this end, 1,642 published fungal genome data were downloaded from published article (Li et al., 2021). We generated a local database and command line makeblastdb -in genome. Fasta -dbtype nucl -out genome was used to perform homology search on the generated local database. The Csn subunits were identified in 1,642 reported fungal genomes by using a BLASTP algorithm, and the sequences of Csn subunits found in *A. nidulans* were used as queries since a complete eight-subunit Csn complex was first identified in this fungus (Busch et al., 2007; Li et al., 2021). Notably, similar to many other fungi, only seven Csn subunits (Csn1-Csn7) were identified in the *F. graminearum* genome (Supplementary Table 2). By searching and screening for sequence homology, we found 8,630 candidate sequences in 1,642 fungal genomes (Figure 1A and Supplementary Table 3). The statistical results showed that each species contained an average of 5.26 Csn proteins; however, these proteins were distributed differently among fungi. Csn2 and Csn5 homologs are present in almost all fungal genomes, while Csn8 is almost absent in branches other than Pezizomycotina (Supplementary Figure 1 and Supplementary Table 3).

To investigate the conservation of the Csn complex proteins in fungi, we compared the similarity of eight Csn subunits in *A. nidulans* with the most homologous sequences in 1,642 fungal species. As shown in Figure 1B, Supplementary Figure 1, and Supplementary Table 3, the average conservation values of these eight Csn subunits varied widely in fungi, ranging from 1.19 to 49.96%. Csn5, Csn2, and Csn4 were the top three conserved proteins in all fungi, with average identities of 49.96, 40.72, and 31.08%, respectively, while Csn8 was the least conserved, with an average identity of 1.19%; thus, Csn8 may be present and function only in specific species.

### 3.2. Protein–protein interactome of the Csn complex in *F. graminearum*

**FIGURE 2:**
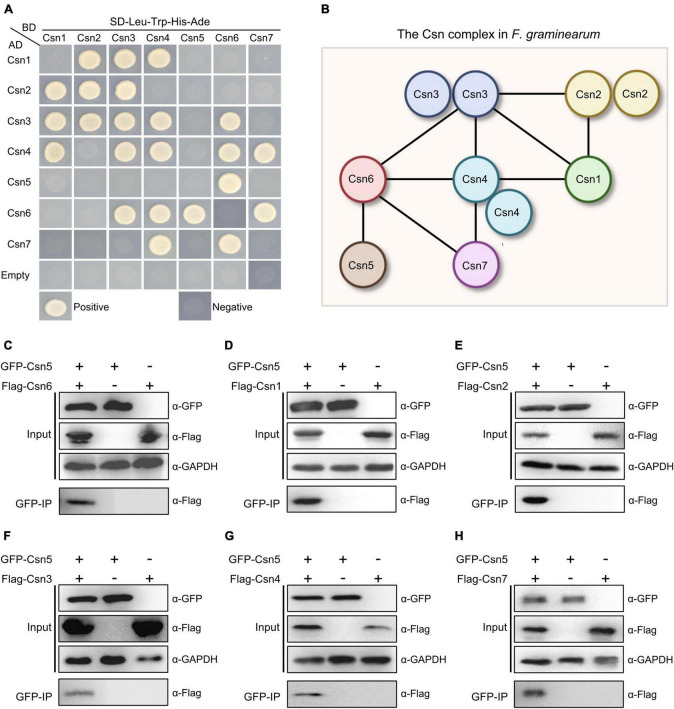
Protein–protein interactome of the Csn complex in *F. graminearum*. **(A)** Interactions between the Csn subunits. The interactions of pGBKT7-53/pGADT7-T and pGBKT7-Lam/pGADT7-T were used as positive and negative controls, respectively. Yeast transformants carrying the indicated constructs were plated onto selective plates supplemented without Leu/Trp/His/Ade to assay growth. **(B)** A model depicting the protein–protein interactions among the Csn subunits of the Csn complex. The black lines indicate the direct interaction. **(C)** Western blot showing the interaction between Csn5 and Csn6 by using a coimmunoprecipitation (co-IP) assay. Total proteins isolated from the strains bearing GFP-Csn5 and/or Flag-Csn6 (input) and the proteins eluted from the anti-GFP beads (elution) were detected using anti-Flag antibody. GAPDH was used as an internal control. **(D)** Western blot showing the interaction between Csn5 and Csn1 by using a co-IP assay. **(E)** Western blot showing the interaction between Csn5 and Csn2 by using a co-IP assay. **(F)** Western blot showing the interaction between Csn5 and Csn3 by using a co-IP assay. **(G)** Western blot showing the interaction between Csn5 and Csn4 by using a co-IP assay. **(H)** Western blot showing the interaction between Csn5 and Csn7 by using a co-IP assay.

To reveal and clarify the molecular interaction network of the Csn complex in *F. graminearum*, binary protein interactions of the Csn subunits were analyzed in this study. For this purpose, full-length individual genes in the Csn complex were cloned into the vector pGADT7 (AD) containing the Gal4 activation domain or pGBKT7 (BD) containing the DNA binding domain, and a yeast two-hybrid (Y2H) assay was performed. Pairs from different combinations of the constructs were co-transformed into *S. cerevisiae* strain AH109 for Y2H assays. As shown in Supplementary Figure 2, all yeast strains transformed with corresponding plasmids grew on SD-Leu-Trp plates, indicating that all the plasmids were successfully transformed into yeast strains. Based on the Y2H assays, we found that Cns2, Cns3 and Cns4 were capable of interacting with themselves, while homodimerization was not detected in the other five proteins (Figures 2A, B). Our abovementioned data showed that Csn5 is the most conserved subunit of the Csn complex across the fungal kingdom. Previous studies have shown that the JAMM motif of Csn5 is necessary for deneddylation activity and that Csn5 is the functional core subunit of the Csn complex (Cope et al., 2002; Zhou et al., 2012). However, the Y2H results showed that Csn5 only directly interacted with Csn6 but not the other five Csn subunits (Figure 2A), implying that Csn5 may associate but not directly interact with others. To test this hypothesis, a strain bearing GFP-Csn5 and Flag-Csn6 was generated, and coimmunoprecipitation (co-IP) assays were conducted. As shown in Figure 2C, Flag-Csn6 fusion proteins could be identified in proteins copurified with GFP-Csn5, further confirming the interaction between Csn5 and Csn6. In addition to Csn6, five other Csn subunits (Csn1, Csn2, Csn3, Csn4, and Csn7) were detected in proteins copurified with Csn5 (Figures 2D–H); thus, these five Csn subunits may associate with Csn5 in *F. graminearum* without directly interacting. In addition to the Csn5-Csn6 interaction, nine other interaction pairs were detected in this study, including Csn1-Csn2, Csn1-Csn3, Csn1-Csn4, Csn2-Csn3, Csn3-Csn4, Csn3-Csn6, Csn4-Csn6, Csn4-Csn7, and Csn6-Csn7 (Figure 2A). Collectively, the protein–protein interactome of the Csn complex was clarified in this study (Figure 2B).

### 3.3. The Csn complex localizes to the nucleus and cytoplasm and is important for fungal development

**FIGURE 3:**
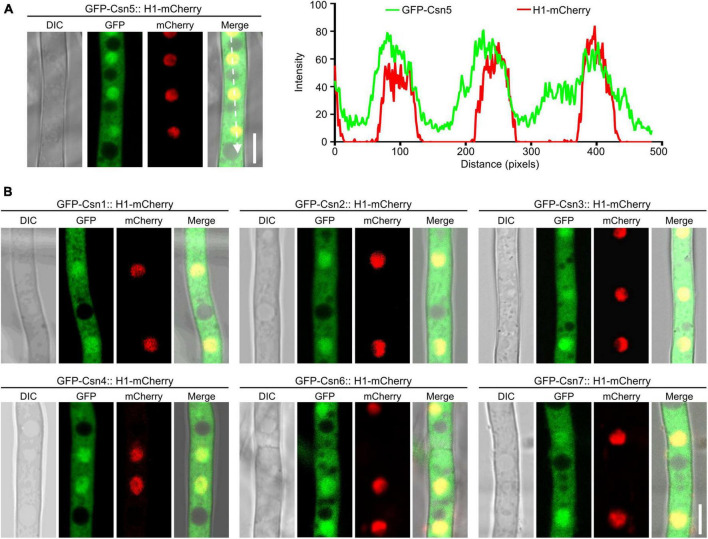
The localization of the Csn subunits in hyphae of *F. graminearum*. **(A)** Micrographs showing the localization of GFP-Csn5 in hyphae of *F. graminearum* after growth in liquid complete medium (CM) for 16 h. H1-mCherry was used to visualize the nuclei. Bar = 5 μm. A line scan graph was generated at the indicated position (arrow) to show the relative localization of GFP-Csn5 (green) and H1-mCherry (red). **(B)** Micrographs showing the localization of six Csn subunits in hyphae of *F. graminearum*. Bar = 5 μm.

Previous studies showed that nuclear localization of Csn subunits has been confirmed in plants, mammals and several fungi, such as *S. cerevisiae*, *S. pombe*, and *Beauveria bassiana* (Kwok et al., 1998; Seeger et al., 2001; Mundt et al., 2002; Maytal-Kivity et al., 2003; Mou et al., 2021). To investigate the localization of the Csn complex in *F. graminearum*, a strain expressing GFP-Csn5 and the nuclear marker histone 1 (H1)-mCherry was generated and the localization of Csn5 was examined under confocal microscopy. As shown in Figure 3A, Csn5-GFP was partially colocalized with H1-mCherry, and certain fluorescence signals were also observed in the cytoplasm, suggesting that Csn5 localized both to the nucleus and cytoplasm. Moreover, the other six subunits of the Csn complex showed similar localization patterns as that of Csn5 (Figure 3B). Collectively, we conclude that the Csn complex localizes to the nucleus and cytoplasm in *F. graminearum*.

**FIGURE 4:**
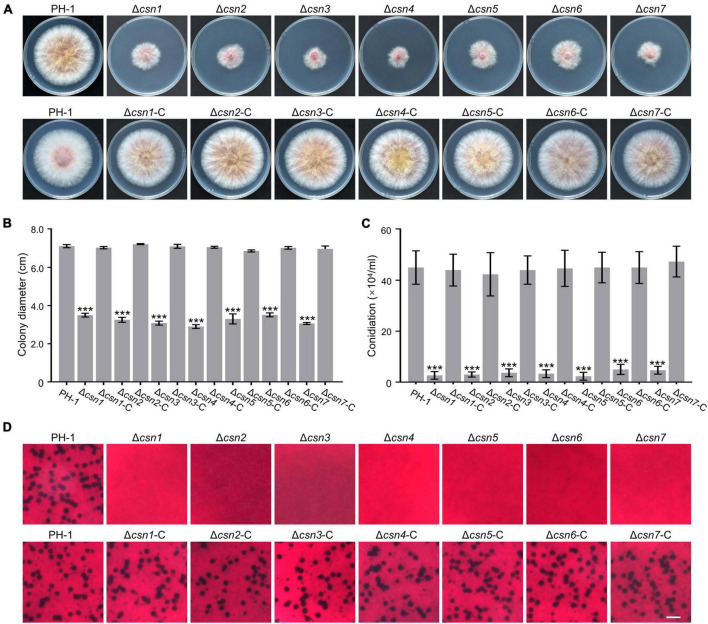
The Csn complex is involved in the fungal development of *F. graminearum*. **(A)** Colony of each strain grown on potato dextrose agar (PDA) medium at 25°C for 3 days. **(B)** Bar charts showing the colony diameters of the indicated strains. **(C)** Bar charts showing the number of conidia produced by the tested strains in liquid CMC medium. **(D)** Micrographs showing the perithecial formation of the indicated strains after growth on carrot agar for 4 weeks. Bar = 1 mm. Error bars indicate the standard deviation from three independent experiments. Statistical analysis was performed via Student’s *t*-test. A triple asterisk indicates statistical significance with *p* < 0.001.

To investigate the role of the Csn complex in *F. graminearum*, a gene replacement approach was used to generate *CSN* gene deletion mutants. For individual *CSN* genes, at least three positive knockout transformants with similar phenotypes, which are described below, were obtained (Supplementary Figure 3). To determine the roles of the Csn complex in hyphal growth, the obtained Csn mutants along with the wild-type strain PH-1 were cultured on potato dextrose agar (PDA) medium at 25°C for 3 days. According to Figures 4A, B, disruption of the Csn complex resulted in a dramatic reduction in hyphal growth compared to that of PH-1. To further confirm that growth defect displayed in the Δ*csn5* mutant was directly caused by Csn5 disruption, the open reading frame (ORF) of *CSN5* driven by its own promoter was transformed into Δ*csn5*, and the complementation strain Δ*csn5*-C was generated. A similar approach was used to generate the corresponding complementation strains for other Csn mutants. Our data showed that all complementation strains exhibited hyphal formation comparable to that of the wild-type strain (Figures 4A, B), suggesting that the Csn complex is a critical regulator of vegetative growth in *F. graminearum.*

The roles of the Csn complex in asexual and sexual reproduction were also determined in this study. According to Figure 4C, the amount of conidia produced by Csn mutants was dramatically reduced compared to that of PH-1. For example, the Δ*csn5* mutant produced 2.33 ± 1.52 × 10^4^ conidia mL^–1^, whereas the wild-type strain produced 15-fold more conidia under the same conditions. These findings suggest that the Csn complex positively regulates the conidiation of *F. graminearum.* In addition, sexual reproduction was compared between the wild type and Csn mutants. As shown in Figure 4D, the wild-type strain and complementation strains formed abundant perithecia after incubation on carrot medium for 4 weeks, whereas Csn mutants were sterile and no perithecia were observed, suggesting that the Csn complex is necessary for perithecium development. Based on the collective results, we conclude that the Csn complex plays an important role in the regulation of asexual and sexual development in *F. graminearum.*

### 3.4. The Csn complex regulates stress responses

**FIGURE 5:**
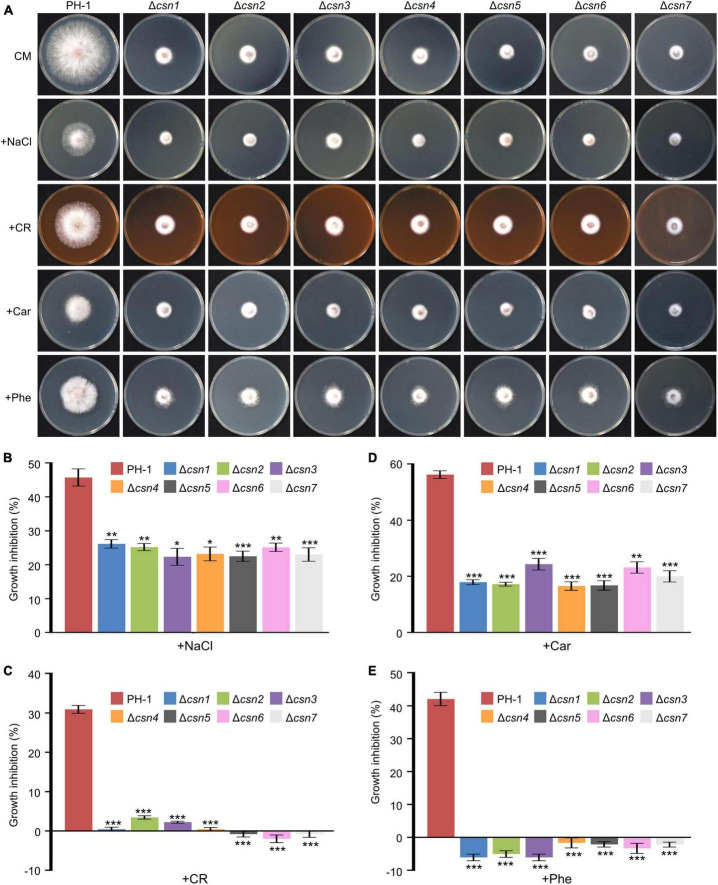
Control of stress responses by the Csn complex. **(A)** Morphologies of the indicated strains after incubation on complete medium (CM) supplemented with multiple abiotic stresses including NaCl, congo red (CR), carbendazim (Car), and phenamacril (Phe). **(B)** The inhibition of the mycelial growth rate was examined after each strain was incubated for 3 days on complete medium supplemented with 1 M NaCl **(B)**, 0.4 g/L congo red **(C)**, 0.5 μg/mL carbendazim **(D)** and 0.25 μg/mL phenamacril **(E)**. The error bars indicate the standard deviation from three independent experiments. Statistical analysis was performed using Student’s *t*-test. The single asterisk indicates statistical significance with *p* < 0.05, the double asterisks indicate statistical significance with *p* < 0.01 and the triple asterisk indicates statistical significance with *p* < 0.001.

To elucidate whether the Csn complex is involved in fungal tolerance to exogenous stress, PH-1 together with Csn mutants were inoculated on complete medium (CM) supplemented with osmotic stress NaCl and cell wall stress Congo red (CR). Our data showed that the loss of the Csn complex led to higher tolerance toward hyperosmotic stress triggered by 1 M NaCl (Figures 5A, B), and the Csn mutants also showed enhanced tolerance for cell wall stress triggered by 0.4 g/L CR (Figures 5A, C). Moreover, the sensitivities of Csn mutants toward fungicides, including carbendazim and phenamacril, were also determined, and we found that Csn mutants also showed better tolerance to these two fungicides than that of PH-1 (Figures 5A, D, E). Moreover, the defects in the stress response displayed in the Csn mutants recovered to the wild-type level in the corresponding complementation strains (Supplementary Figure 4), suggesting that the Csn complex is important for responding to various environmental stresses in *F. graminearum*.

### 3.5. Transcriptome profiling of the wild-type strain and Δ*csn5* mutant

**FIGURE 6:**
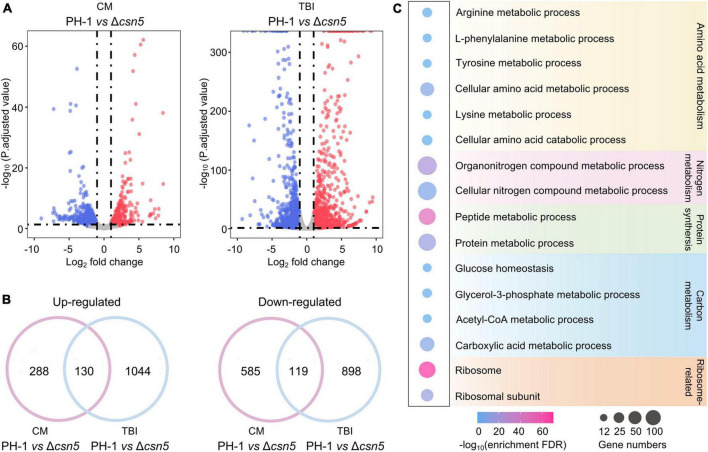
Transcriptome profiles governed by Csn5 in *F. graminearum.* **(A)** Volcano plots showing genes in which the expression was significantly upregulated (red-colored) and downregulated (blue-colored) in the Δ*csn5* mutant compared to the wild-type strain PH-1 (*P* < 0.05, fold change ≥2.0) under nutrient-rich conditions [complete medium (CM)] or toxin induction conditions [trichothecene biosynthesis induction (TBI) medium]. **(B)** Venn diagrams showing the number of genes in which the expression was significantly upregulated (left panel) or downregulated (right panel) in the Δ*csn5* mutant compared with the wild-type strain PH-1 under both nutrient-rich conditions and toxin induction conditions. **(C)** Enriched gene ontology (GO) terms in genes with downregulated transcripts in the Δ*csn5* mutant under nutrient-rich conditions.

Previous findings showed that Csn5 is the functional core subunit of the Csn complex (Cope et al., 2002; Zhou et al., 2012). To explore the underlying mechanisms of the Csn complex in regulating growth and fungal development, total RNA of the wild-type and Δ*csn5* mutant were isolated after incubation in complete medium (CM) (nutrient-rich condition) and trichothecene biosynthesis induction (TBI) medium (toxin induction condition), respectively, and transcriptome profiling was analyzed. Differentially expressed gene (DEG) analysis showed that the expression levels of 1,122 (704 downregulated and 418 upregulated genes) and 2,191 (1,174 downregulated and 1,017 upregulated genes) genes were altered by ≥2.0-fold at *P*-value < 0.05 under nutrient-rich conditions and toxin induction conditions, respectively, in response to the deletion of Csn5 (Figure 6A and Supplementary Table 4). Among these differentially expressed genes, 130 genes were upregulated and 119 genes were downregulated in the Δ*csn5* mutant under both nutrient-rich conditions and toxin induction conditions (Figure 6B). These results suggested that Csn5 might serve as a transcriptional regulator in *F. graminearum.*

Ribosomal proteins are important for ribosome assembly and the biosynthesis of proteins. Previous findings have demonstrated that abnormal expression of ribosomal proteins is associated with defects in growth and development in animals, plants and fungi (Narla and Ebert, 2010; Horiguchi et al., 2012; Wang et al., 2021). Many ribosome-related genes were significantly downregulated with the loss of Csn5 under nutrient-rich conditions (Figure 6C), which was consistent with our findings that Csn5 is crucial for the vegetative growth and fungal development of *F. graminearum*. Moreover, DEG analysis showed that many Csn5-dependent genes were involved in amino acid metabolism, protein synthesis, and carbon and nitrogen metabolism, further supporting the critical role of Csn5 in growth and development. Taken together, the results indicate that Csn5 may regulate hyphal growth and fungal development by regulating the expression levels of genes associated with the assembly of the ribosome, protein synthesis and nutrition metabolism.

### 3.6. The Csn complex mediates DON production by regulating the transcription levels of *TRI* genes

**FIGURE 7:**
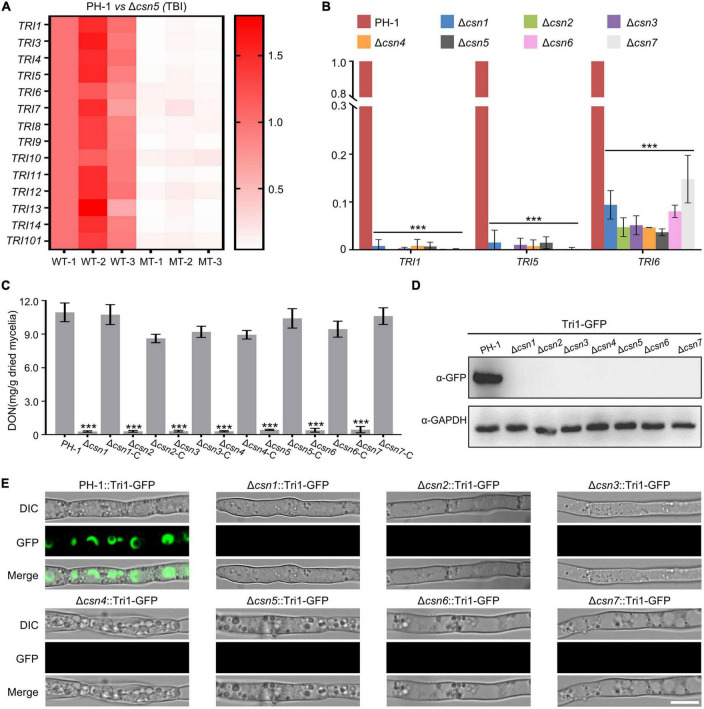
Regulation of DON production by the Csn complex. **(A)** Heatmap comparing the relative transcriptional abundance of *TRI* genes in the wild-type strain or Δ*csn5* mutant in three independent replicates using transcriptomic analysis. WT, wild type. MT = Δ*csn5* mutant. The transcription levels of *TRI* genes in the replicate 1 of the wild-type strain were set to 1.0. **(B)** Bar charts showing the relative transcriptional abundance of *TRI* genes in the wild-type strain or indicated mutant strains using quantitative real-time PCR (qRT–PCR). **(C)** Bar charts showing DON production in 7-day-old TBI cultures of the indicated strains. **(D)** Western blot analysis of Tri1 protein levels in tested strains expressing Tri1-GFP with the anti-GFP antibody. GAPDH was used as an internal control. **(E)** Fluorescence signals of Tri1-GFP were detected in the tested strains after growth in liquid TBI medium for 48 h. Bar = 10 μm. Error bars indicate the standard deviation from three independent experiments. Statistical analysis was performed via Student’s *t*-test. A triple asterisk indicates statistical significance with *p* < 0.001.

As the key virulence factor, the mycotoxin DON produced by *F. graminearum* is synthesized in a specific cellular region called the toxisome, which contains trichothecene synthase and is mainly encoded by the 14 *TRI* genes (Alexander et al., 2009; Tang et al., 2018; Chen et al., 2019). DEG analysis showed that the expression levels of all 14 *TRI* genes were dramatically reduced after the loss of Csn5 under toxin induction conditions (Figure 7A and Supplementary Table 4), indicating that the Csn5 positively regulated the expression of DON biosynthesis-related genes. To confirm the transcriptome results, the transcription levels of three *TRI* genes (*TRI1*, *TRI5*, and *TRI6*) randomly selected from 14 *TRI* genes were determined using quantitative real-time PCR (qRT–PCR). In agreement with the transcriptome data, the transcript abundance of these three trichothecene biosynthesis-related genes was significantly downregulated with the loss of Csn5 (Figure 7B). Since the deletion of Csn5 leads to dramatically reduced transcription levels of DON biosynthesis-related genes, DON production in Δ*csn5* and PH-1 was determined in this study. Consistently, our results revealed that the Δ*csn5* mutant produced approximately 25-fold less DON than that of PH-1 (Figure 7C). Tri1 is a calmodulin oxygenase that functions at a late stage of DON biosynthesis (McCormick et al., 2004). To further confirm the impact of Csn5 on toxin production, the wild-type strain and Δ*csn5* mutant expressing Tri1-GFP were generated, and toxisome formation was determined in this study. Immunoblot analysis revealed that the Tri1-GFP level was negligible in the mutant, as determined with an anti-GFP antibody (Figure 7D). In agreement with the immunoblot analysis, Tri1-GFP was almost undetected after the deletion of Csn5, whereas strong fluorescence signals were found in spherical- or crescent-shaped toxisomes in the wild-type background after 48 h of incubation in TBI medium (Figure 7E). Similar to Csn5, we demonstrated that the other Csn subunits were also necessary for the transcription of DON biosynthesis-related genes, toxisome formation and DON production (Figures 7B–E). Taken together, our data suggest that the Csn complex is necessary for the toxisome formation and DON production in *F. graminearum*.

### 3.7. The Csn complex is critical for fungal infection

**FIGURE 8:**
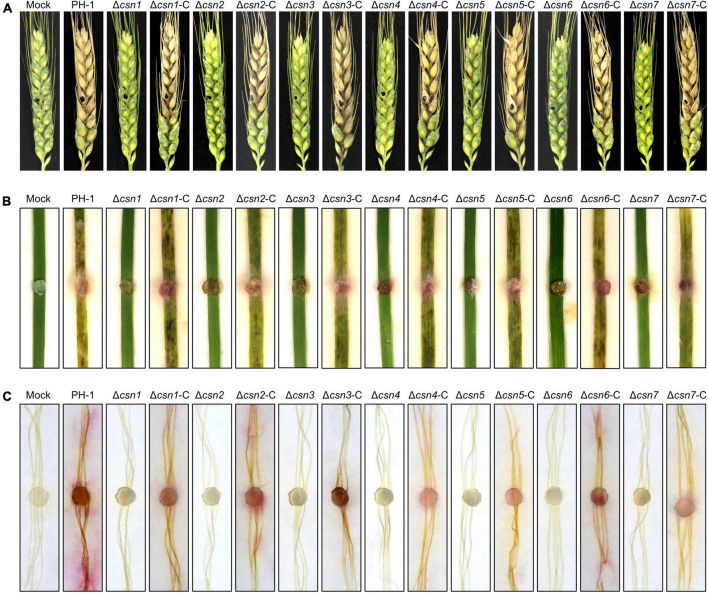
The Csn complex is necessary for *F. graminearum* virulence. **(A)** Flowering wheat heads were inoculated with mycelial plugs from the indicated strains, and photos were taken 14 days post-inoculation (dpi). The mycelial plugs of the indicated strains were used to inoculate wheat leaves **(B)** and corn silks **(C)**, and disease symptoms were recorded after 7 dpi.

Previous studies revealed that the absence of genes necessary for environmental stress (e.g., osmotic and cell wall stresses) responses always impaired the full virulence of *F. graminearum* (Hou et al., 2002; Wang et al., 2011; Zheng et al., 2012; Yun et al., 2014), suggesting that appropriate responses to environmental stimuli are critical for fungal virulence. Moreover, the mycotoxin DON produced by *F. graminearum* is an important virulence factor in host plants. Since our data showed that the disruption of the Csn complex impaired the normal response to environmental stresses and DON production, the effects of the Csn complex on *F. graminearum* virulence were tested on flowering wheat heads. According to Figure 8A, we found that the depletion of the individual Csn subunit led to the loss of *F. graminearum* virulence. No visible disease was caused by the Csn mutants on inoculated wheat spikelets, but typical scab symptoms were detected in spikelets inoculated with PH-1 or corresponding complementation strains under identical conditions. To further confirm the effect of the Csn complex on *F. graminearum* virulence, the virulence of Csn mutants on wheat leaves and corn silks was also examined. Consistently, Csn mutants did not cause any detectable symptoms (Figures 8B, C), suggesting that the Csn complex is important for *F. graminearum* virulence, which is tissue- and host-independent.

## 4. Discussion

The Csn complex is an evolutionarily conserved complex that controls multiple cellular and developmental processes in plants, mammals and fungi (Kato and Yoneda-Kato, 2009; Braus et al., 2010; Qin et al., 2020). Compared to plants and mammals, in fungi, the functions and roles of the Csn complex remain poorly understood, and our current knowledge about how this complex regulates fungal development and virulence is limited. In this study, detailed bioinformatic, phenotypic, genetic, live cell imaging, and biochemical approaches provided a comprehensive overview of the possible biological functions of the *F. graminearum* Csn complex in fungal development and virulence.

For the seven subunits of the Csn complex in *F. graminearum*, the pairwise interactions were examined using a yeast two-hybrid assay, and we found that self-interactions of Csn2, Csn3, and Csn4 were detected from this comprehensive assay (Figure 2A), suggesting that these three subunits could form dimes or mediate potential Csn-Csn interactions. Csn4 was able to interact with itself, which was consistent with previous data for *A. nidulans*, mammalian and *Arabidopsis*, while the self-interaction of Csn2 was also detected in *A. nidulans* and mammalian but not *Arabidopsis* (Serino et al., 2003; Busch et al., 2007). Some organism-specific self-interaction patterns were also detected; for example, Csn1 was capable of interacting with itself in *Arabidopsis* but not in other organisms, while homodimerization of Csn6 was detected only in *A. nidulans* (Serino et al., 2003; Busch et al., 2007). In addition to self-interactions, 10 interacting pairs were identified in this study, while 7, 12, and 19 interacting pairs were detected in *A. nidulans*, mammals and *Arabidopsis*, respectively, suggesting that the interaction patterns might be more complex in mammals and plants than in fungi. Several interactions, including Csn1-Csn2, Csn1-Csn4, Csn4-Csn7, Csn4-Csn6, and Csn5-Csn6, were all demonstrated in these organisms (Serino et al., 2003; Busch et al., 2007). Taken together, these results indicate that even though there may be some differences in the interacting pairs of the Csn complex among *A. nidulans*, mammalian, *Arabidopsis* and *F. graminearum*, the basic interaction network seems to be conserved.

Asexual reproduction is of great importance in the *F. graminearum* life cycle since conidia are necessary for colonization and transmission of disease (Trail et al., 2002; Chen et al., 2022). One of the marked defects displayed in the Csn mutants was that the number of conidia produced by the Csn mutants was dramatically reduced in comparison to that of the wild-type strain (Figure 4C). Consistently, all the Csn mutants except for the Csn3 mutant produced fewer conidia in *N. crassa* (He et al., 2005; Wang et al., 2010). The deletion of Csn6 in *M. oryzae* and Csn5 in *Alternaria alternata* and *Beauveria bassiana* also led to a reduction in asexual sporulation (Wang et al., 2018; Mou et al., 2021, Shen et al., 2022). However, Csn was not important for conidiation in *A. nidulans* (Busch et al., 2003; Nahlik et al., 2010), suggesting that the roles of Csn in regulating asexual development are species specific.

Previous studies showed that the loss of Csn6 in *Magnaporthe oryzae* and Csn5 in *A. alternata* were associated with fungal virulence (Wang et al., 2018; Shen et al., 2022). To our knowledge, except for these two individual subunits of the Csn complex in *M. oryzae* and *A. alternata*, little is known about the biological functions of the Csn complex in fungal virulence in plant fungal pathogens. In this study, systematic functional characterization of the Csn complex was performed, and we found that the deleting every Csn subunit led to the loss of *F. graminearum* virulence on host plants, including wheat and corn, which may be attributed to several regulatory mechanisms. First, the growth rate of the Csn mutant decreased by approximately 50% in comparison to that of the wild-type strain, which partly contributed to the inability to infect the plants. Second, an increasing number of studies have revealed that proper responses to environmental stimuli are crucial for fungal virulence (Hou et al., 2002; Wang et al., 2011; Zheng et al., 2012; Yun et al., 2014). For example, the cell wall intergrity (CWI) pathway is required for responding to cell wall stress signals. Previous studies have shown that the disruption of Bck1, Mkk1, and Mgv1, the core components of the CWI pathway, led to the loss of virulence (Hou et al., 2002; Wang et al., 2011; Yun et al., 2014). In this study, our results demonstrated that Csn mutants enhanced resistance to hyperosmotic stress of NaCl; Congo red, a cell wall damaging agent; and fungicides, including carbendazim and phenamacril. Therefore, an abnormal stress response to environmental cues could be among the reasons for the reduced virulence displayed by the Csn mutants. Third, the mycotoxin DON secreted by *F. graminearum* is an important virulence factor that plays a vital function of mediating fungal spreading in host plants. Our data showed that the transcriptional abundance of *TRI* genes, toxisome formation and DON production were dramatically reduced with the deletion of the Csn complex, which largely prevented the spread of the fungus within host tissues. Taken together, these findings present new insights into the function of the Csn complex in regulating fungal virulence, providing novel possibilities and targets to control FHB.

## 5. Conclusion

**FIGURE 9:**
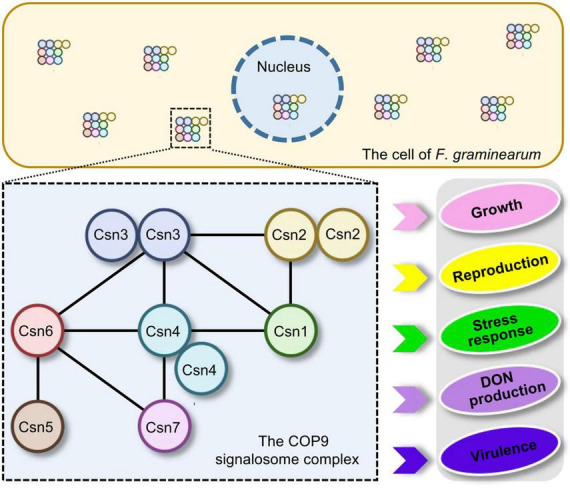
Proposed model for the regulation of fungal development and virulence by the Csn complex in *F. graminearum*. The dashed box represents the Csn complex and its interactions. The black lines within the box indicate the direct interactions between the Csn subunits. The Csn complex localizes to the nucleus and cytoplasm and the loss of the Csn complex leads to defects in hyphal growth, asexual and sexual reproduction, stress response, DON production and virulence.

In conclusion, the distribution of the Csn subunit in the fungal kingdom was surveyed and the interaction among Csn subunits was clarified in *F. graminearum*. Moreover, a systematic functional characterization of the Csn complex was performed in this study. Our data revealed that the Csn complex localized to the nucleus and cytoplasm and that the Csn subunits function together in controlling a wide range of developmental processes in *F. graminearum*, including growth, asexual and sexual reproduction, stress response, DON production and virulence (Figure 9). Given the fundamental function of the Csn complex in mediating deneddylation, it would be interesting to determine its links to other post-translational modifications. More importantly, further identification and functional characterization of substrates regulated by the Csn complex should further clarify the Csn function in *F. graminearum*, especially in fungal virulence, providing novel possibilities and targets to control FHB.

## Data availability statement

The data presented in this study are deposited in the National Center for Biotechnology Information (NCBI) Sequence Read Archive (SRA), accession number: PRJNA947915.

## Author contributions

YC, ZM, and AC conceived and designed the study, analyzed the data, and wrote the manuscript. AC, YR, XH, CL, YZ, CX, and HQ performed the experiments. All authors read and approved the final version of the manuscript.

## Supplementary material

Supplementary Figure 1 Evolutionary conservation of the Csn subunits in fungi.

Supplementary Figure 2 Yeast transformants carrying the indicated constructs were plated onto selective plates supplemented without Leu/Trp to assay growth.

Supplementary Figure 3 Targeted gene deletion of the Csn complex in *F. graminearum*. **(A)** The Csn subunits and *HPH* genes are marked with large green and blue arrows, respectively. PCR primers are marked with small arrows. **(B)** Deletion mutants were identified by PCR assays. The size of the PCR products is indicated. **(C)** The DNA marker used in S3 **(B)**.

Supplementary Figure 4 The defect in the stress response was fully recovered to the wild-type level in the complementation strains of Csn mutants. **(A)** Morphologies of the indicated strains after incubation on complete medium (CM) supplemented with multiple abiotic stresses including NaCl, Congo red (CR), carbendazim (Car), and phenamacril (Phe). **(B)** The inhibition of the mycelial growth rate was examined after each strain was incubated for 3 days on complete medium supplemented with 1 M NaCl (B), 0.4 g/L Congo red (C), 0.5 μg/mL carbendazim (D) and 0.25 μg/mL phenamacril (E). Error bars indicate the standard deviation from three independent experiments.
