# Supplementary material for: The COP9 signalosome complex regulates fungal development and virulence in the wheat scab fungus Fusarium graminearum
Source: Front Microbiol. 2023 Apr 24;14:1179676. doi: 10.3389/fmicb.2023.1179676 (PMC10165099; doi:10.3389/fmicb.2023.1179676)
Supplement: Supplementary file 1 [file Table_1.DOCX]

**Table S1. List of primers used in this study**

| Primer | Sequence(5’-3’) | Relevant Characteristics |
| --- | --- | --- |
| Csn1-Up-F | GCGTCTTCGGGCTTTTGT | PCR primers for amplification of the upstream fragment of *CSN1* |
| Csn1-Up-R | TTGACCTCCACTAGCTCCAGCCAAGCCGCTCCCAGTTCACCTCTCA |  |
| Csn1-Down-F | GAATAGAGTAGATGCCGACCGCGGGTTTAGATCAGGCGTCCAGGA | PCR primers for amplification of the downstream fragment of *CSN1* |
| Csn1-Down-R | CAAAGGATAAGGAGGGTAGG |  |
| Csn1-Ex-F | CGTCAAGTTACAGCTCTC | PCR primers for the external identification of *CSN1* disruption |
| Csn1-Ex-R | GGTAATTGTGTGAAGAGC |  |
| Csn1-In-F | CAGAATGGGTTGATCGAG | PCR primers for the inner identification of *CSN1* disruption |
| Csn1-In-R | GTTCTTTGCATCCAGGTG |  |
| Csn2-Up-F | TGGTCTTTGGAACGCTGT | PCR primers for amplification of the upstream fragment of *CSN2* |
| Csn2-Up-R | TTGACCTCCACTAGCTCCAGCCAAGCCGTGAATGAGGTGTGGTGGA |  |
| Csn2-Down-F | GAATAGAGTAGATGCCGACCGCGGGTTCGCAAATCGTCCGTCTCG | PCR primers for amplification of the downstream fragment of *CSN2* |
| Csn2-Down-R | GCACTTCTCGCAAGGTCAC |  |
| Csn2-Ex-F | GCTCCACATCAGCTTCTG | PCR primers for the external identification of *CSN2* disruption |
| Csn2-Ex-R | CCAGGTAACAAGATTTGC |  |
| Csn2-In-F | GCAGGCCATAAAACTTG | PCR primers for the inner identification of *CSN2* disruption |
| Csn2-In-R | CGAGTCGTCCAGAATG |  |
| Csn3-Up-F | GAAAGGAGGTCTGGAGTAGCG | PCR primers for amplification of the upstream fragment of *CSN3* |
| Csn3-Up-R | TTGACCTCCACTAGCTCCAGCCAAGCCAAGTTGCCGTCACAGCGTC |  |
| Csn3-Down-F | GAATAGAGTAGATGCCGACCGCGGGTTTAGAAAGCCAGGTTATCGG | PCR primers for amplification of the downstream fragment of *CSN3* |
| Csn3-Down-R | GTTAAGGTAAAGTCGTCCCC |  |
| Csn3-Ex-F | CATCATCATCATCGGCCTC | PCR primers for the external identification of *CSN3* disruption |
| Csn3-Ex-R | CCGCAATGCCACAACTAC |  |
| Csn3-In-F | CGCATTTCTAGCCATCC | PCR primers for the inner identification of *CSN3* disruption |
| Csn3-In-R | GATTAGAGACGAGGTACC |  |
| Csn4-Up-F | TCATCGTCGCCAGAAGTC | PCR primers for amplification of the upstream fragment of *CSN4* |
| Csn4-Up-R | TTGACCTCCACTAGCTCCAGCCAAGCCGAACGGGGAGTGGAAGTG |  |
| Csn4-Down-F | GAATAGAGTAGATGCCGACCGCGGGTTTTTCTCGTGTATGGCGTTTT | PCR primers for amplification of the downstream fragment of *CSN4* |
| Csn4-Down-R | TCATTGCGACTCCTTCCG |  |
| Csn4-Ex-F | CTATACCCCACTAGAGC | PCR primers for the external identification of *CSN4* disruption |
| Csn4-Ex-R | CAGCGATGGATATGATGG |  |
| Csn4-In-F | GCACTTCGAGAGATAGAG | PCR primers for the inner identification of *CSN4* disruption |
| Csn4-In-R | CCTTTTGCAACGCGTTGG |  |
| Csn5-Up-F | GGATTTGGCTTTTTTACTGC | PCR primers for amplification of the upstream fragment of *CSN5* |
| Csn5-Up-R | TTGACCTCCACTAGCTCCAGCCAAGCCGGATGCCTCGTCTTATTGAT |  |
| Csn5-Down-F | GAATAGAGTAGATGCCGACCGCGGGTTTCTTTTAGACAACTTGCTCCG | PCR primers for amplification of the downstream fragment of *CSN5* |
| Csn5-Down-R | CAACACACCCCTTCTCACG |  |
| Csn5-Ex-F | GGTTCCTATTCTCTCTATGG | PCR primers for the external identification of *CSN5* disruption |
| Csn5-Ex-R | CCAAGACATTGGCTAATC |  |
| Csn5-In-F | CGCTCTTCGCTATCAGTAC | PCR primers for the inner identification of *CSN5* disruption |
| Csn5-In-R | CTGGCCTTGACTTGGTTC |  |
| Csn6-Up-F | GACAAAACCAGAGACGGATT | PCR primers for amplification of the upstream fragment of *CSN6* |
| Csn6-Up-R | TTGACCTCCACTAGCTCCAGCCAAGCCGTGAGTTGAGGTGGATTGATAA |  |
| Csn6-Down-F | GAATAGAGTAGATGCCGACCGCGGGTTCGAGAAGTCTACTAGCCGAAG | PCR primers for amplification of the downstream fragment of *CSN6* |
| Csn6-Down-R | CTACGCTGTGAGCCAAGG |  |
| Csn6-Ex-F | GGAGCTGGAGCTAATG | PCR primers for the external identification of *CSN6* disruption |
| Csn6-Ex-R | GATGCTTATCAACTGTTC |  |
| Csn6-In-F | GACTATCCTCCCCATCCAC | PCR primers for the inner identification of *CSN6* disruption |
| Csn6-In-R | GATGGCCAGCCTTTGCAG |  |
| Csn7-Up-F | TTGAGGAGCACACTACCATT | PCR primers for amplification of the upstream fragment of *CSN7* |
| Csn7-Up-R | TTGACCTCCACTAGCTCCAGCCAAGCCTCGTGATTGAGGCTACAGC |  |
| Csn7-Down-F | GAATAGAGTAGATGCCGACCGCGGGTTATAATAGATGACACGACGCC | PCR primers for amplification of the downstream fragment of *CSN7* |
| Csn7-Down-R | CACGATATCACCACCGAA |  |
| Csn7-Ex-F | CAGCACTGGTCCTTACAG | PCR primers for the external identification of *CSN7* disruption |
| Csn7-Ex-R | GTCAATAGGGCTGCCG |  |
| Csn7-In-F | CGATTGCAGCCCTTCCTC | PCR primers for the inner identification of *CSN7* disruption |
| Csn7-In-R | CATGCTGTCGTTGTCCACC |  |
| *HPH*-F | GGAGGTCAACACATCAATGCCTATT | PCR primers to amplify fragment of *HPH* |
| *HPH*-R | CTACTCTATTCCTTTGCCCT |  |
| Csn1-AD-F | GTACCAGATTACGCTCATATGATGTCCGAATCAAACGCTC | PCR primers to amplify full *CSN1* for construction of pGADT7-Csn1 |
| Csn1-AD-R | ACTGGCCTCCATGGCCATATGAACTCCTTCAATTTGGC |  |
| Csn1-BD-F | TCAGAGGAGGACCTGCATATGATGTCCGAATCAAACGCTC | PCR primers to amplify full *CSN1* for construction of pGBKT7-Csn1 |
| Csn1-BD-R | TTCGGCCTCCATGGCCATATGAACTCCTTCAATTTGGC |  |
| Csn2-AD-F | GTACCAGATTACGCTCATATGATGTCAGACGACGAGG | PCR primers to amplify full *CSN2* for construction of pGADT7-Csn2 |
| Csn2-AD-R | ACTGGCCTCCATGGCCATATGGCCCAAGCCGCTGCGGC |  |
| Csn2-BD-F | TCAGAGGAGGACCTGCATATGATGTCAGACGACGAGG | PCR primers to amplify full *CSN2* for construction of pGBKT7-Csn2 |
| Csn2-BD-R | TTCGGCCTCCATGGCCATATGGCCCAAGCCGCTGCGGC |  |
| Csn3-AD-F | GTACCAGATTACGCTCATATGATGGTAGTGACACGTGCG | PCR primers to amplify full *CSN3* for construction of pGADT7-Csn3 |
| Csn3-AD-R | ACTGGCCTCCATGGCCATATGAACATCGTGTTTCGACTTGGCC |  |
| Csn3-BD-F | TCAGAGGAGGACCTGCATATGATGGTAGTGACACGTGCG | PCR primers to amplify full *CSN3* for construction of pGBKT7-Csn3 |
| Csn3-BD-R | TTCGGCCTCCATGGCCATATGAACATCGTGTTTCGACTTGGCC |  |
| Csn4-AD-F | GTACCAGATTACGCTCATATGATGACGCCCAGCCCTG | PCR primers to amplify full *CSN4* for construction of pGADT7-Csn4 |
| Csn4-AD-R | ACTGGCCTCCATGGCCATATGTTATACAACAAGGTTTGCGGC |  |
| Csn4-BD-F | TCAGAGGAGGACCTGCATATGATGACGCCCAGCCCTG | PCR primers to amplify full *CSN4* for construction of pGBKT7-Csn4 |
| Csn4-BD-R | TTCGGCCTCCATGGCCATATGTTATACAACAAGGTTTGCGGC |  |
| Csn5-AD-F | GTACCAGATTACGCTCATATGATGGAGGCATCAGCTCTC | PCR primers to amplify full *CSN5* for construction of pGADT7-Csn5 |
| Csn5-AD-R | ACTGGCCTCCATGGCCATATGTCATTCCGACGTAGGGTTCGCC |  |
| Csn5-BD-F | TCAGAGGAGGACCTGCATATGATGGAGGCATCAGCTCTC | PCR primers to amplify full *CSN5* for construction of pGBKT7-Csn5 |
| Csn5-BD-R | TTCGGCCTCCATGGCCATATGTCATTCCGACGTAGGGTTCGCC |  |
| Csn6-AD-F | GTACCAGATTACGCTCATATGATGACTACCACAACCACG | PCR primers to amplify full *CSN6* for construction of pGADT7-Csn6 |
| Csn6-AD-R | ACTGGCCTCCATGGCCATATGTTCTTTGTAATGTTTTTTGC |  |
| Csn6-BD-F | TCAGAGGAGGACCTGCATATGATGACTACCACAACCACG | PCR primers to amplify full *CSN6* for construction of pGBKT7-Csn6 |
| Csn6-BD-R | TTCGGCCTCCATGGCCATATGTTCTTTGTAATGTTTTTTGC |  |
| Csn7-AD-F | GTACCAGATTACGCTCATATGATGGAACAGACAAAGGC | PCR primers to amplify full *CSN7* for construction of pGADT7-Csn7 |
| Csn7-AD-R | ACTGGCCTCCATGGCCATATGTCACAGCTTGCGCTTGCTCG |  |
| Csn7-BD-F | TCAGAGGAGGACCTGCATATGATGGAACAGACAAAGGC | PCR primers to amplify full *CSN7* for construction of pGBKT7-Csn7 |
| Csn7-BD-R | TTCGGCCTCCATGGCCATATGTCACAGCTTGCGCTTGCTCG |  |
| Csn8-AD-F | GTACCAGATTACGCTCATATGATGAACACTACTTTCCAACAG | PCR primers to amplify full *CSN8* for construction of pGADT7-Csn8 |
| Csn8-AD-R | ACTGGCCTCCATGGCCATATGTCACACCTGTCCCGG |  |
| Csn8-BD-F | TCAGAGGAGGACCTGCATATGATGAACACTACTTTCCAACAG | PCR primers to amplify full *CSN8* for construction of pGBKT7-Csn8 |
| Csn8-BD-R | TTCGGCCTCCATGGCCATATGTCACACCTGTCCCGG |  |
| AD-ID-F | TAATACGACTCACTATAGGGCGAGC | PCR primers to identify the positive clones in yeast transformation |
| AD-ID-R | AGATGGTGCACGATGCACA |  |
| Csn1-Native-pro-F | TTACTGGTGGAAAAGGTGC | PCR primers to amplify the native promoter of *CSN1* gene |
| Csn1-Native-pro-R | ACAGCTCCTCGCCCTTGCTCACCAT TATAGATGTTAAGCTTATCTGC |  |
| GFP-F | ATGGTGAGCAAGGGCGA | PCR primers to amplify the GFP |
| GFP-R | CTTGTACAGCTCGTCCATGC |  |
| GFP-Csn1-ORF-F | TCTCGGCATGGACGAGCTGTACAAG ATGTCCGAATCAAACGCTCT | PCR primers to amplify the ORF of *CSN1* gene for the construction of GFP-Csn1 vector |
| GFP-Csn1-ORF-R | GCTACACGATGGTGAAAAGG |  |
| Csn2-Native-pro-F | CCGACGAGGATAACACTTACG | PCR primers to amplify the native promoter of *CSN2* gene |
| Csn2-Native-pro-R | ACAGCTCCTCGCCCTTGCTCACCAT GGTGAATGAGGTGTGGTGGA |  |
| GFP-Csn2-ORF-F | TCTCGGCATGGACGAGCTGTACAAG ATGTCAGACGACGAGGATTT | PCR primers to amplify the ORF of *CSN2* gene for the construction of GFP-Csn2 vector |
| GFP-Csn2-ORF-R | ACAGGCTCTATGGTTCTTGTAC |  |
| Csn3-Native-pro-F | GTGACAGTGACGAGGATACGG | PCR primers to amplify the native promoter of *CSN3* gene |
| Csn3-Native-pro-R | ACAGCTCCTCGCCCTTGCTCACCAT TCTGAGACCAATCGCGGAT |  |
| GFP-Csn3-ORF-F | TCTCGGCATGGACGAGCTGTACAAG ATGGACGCTGTGACGGCA | PCR primers to amplify the ORF of *CSN3* gene for the construction of GFP-Csn3 vector |
| GFP-Csn3-ORF-R | AGATGGTGGGTAGGGGCTGT |  |
| Csn4-Native-pro-F | ATCAGTCAACCAGCAGATCG | PCR primers to amplify the native promoter of *CSN4* gene |
| Csn4-Native-pro-R | ACAGCTCCTCGCCCTTGCTCACCAT TGTTGTTTCGTGGTGAGATT |  |
| GFP-Csn4-ORF-F | TCTCGGCATGGACGAGCTGTACAAG ATGACGCCCAGCCCTGAA | PCR primers to amplify the ORF of *CSN4* gene for the construction of GFP-Csn4 vector |
| GFP-Csn4-ORF-R | CTCGTGGCCGATACGAAGAG |  |
| Csn5-Native-pro-F | ACAGGCGCATTACTCGG | PCR primers to amplify the native promoter of *CSN5* gene |
| Csn5-Native-pro-R | ACAGCTCCTCGCCCTTGCTCACCAT TGTGACGACCAAAGATGTTG |  |
| GFP-Csn5-ORF-F | TCTCGGCATGGACGAGCTGTACAAG ATGGAGGCATCAGCTCTCAA | PCR primers to amplify the ORF of *CSN5*gene for the construction of GFP-Csn5 vector |
| GFP-Csn5-ORF-R | CACCCGCTACAAGAACAAGA |  |
| Csn6-Native-pro-F | GAGGGTTGGAGATTCAAGG | PCR primers to amplify the native promoter of *CSN6* gene |
| Csn6-Native-pro-R | ACAGCTCCTCGCCCTTGCTCACCAT AACGAACGAGCTGCGAGG |  |
| GFP-Csn6-ORF-F | TCTCGGCATGGACGAGCTGTACAAG ATGACTACCACAACCACGAC | PCR primers to amplify the ORF of *CSN6* gene for the construction of GFP-Csn6 vector |
| GFP-Csn6-ORF-R | CTTGACAAGGCACAGGCT |  |
| Csn7-Native-pro-F | AGGATGTTCCAGAAGGGTC | PCR primers to amplify the native promoter of *CSN7* gene |
| Csn7-Native-pro-R | ACAGCTCCTCGCCCTTGCTCACCAT GATGAGTCGTGATTGAGGCT |  |
| GFP-Csn7-ORF-F | TCTCGGCATGGACGAGCTGTACAAG ATGGAACAGACAAAGGCTCT | PCR primers to amplify the ORF of *CSN7* gene for the construction of GFP-Csn7 vector |
| GFP-Csn7-ORF-R | GACTTTTCTCGTCGATGCC |  |
| Flag-Csn1-F | CATGATATCGATTACAAGGATGACGATGACAAGATGTCCGAATCAAACGCTC | PCR primers to amplify *CSN1* gene for the construction of Flag-Csn1 vector |
| Flag-Csn1-R | CGCAGAGGAGCCTGAATGTTGAGTGGAATGATGCTAAACTCCTTCAATTTGGC |  |
| Flag-Csn2-F | CATGATATCGATTACAAGGATGACGATGACAAGATGTCAGACGACGAGG | PCR primers to amplify *CSN2* gene for the construction of Flag-Csn2 vector |
| Flag-Csn2-R | CGCAGAGGAGCCTGAATGTTGAGTGGAATGATGCAAGCCCAAGCCGCTGC |  |
| Flag-Csn3-F | CATGATATCGATTACAAGGATGACGATGACAAGATGGACGCTGTGACGGCAACTT | PCR primers to amplify *CSN3* gene for the construction of Flag-Csn3 vector |
| Flag-Csn3-R | CGCAGAGGAGCCTGAATGTTGAGTGGAATGATGCTAGCCATGTGCCATGATGCC |  |
| Flag-Csn4-F | CATGATATCGATTACAAGGATGACGATGACAAGATGACGCCCAGCCCTG | PCR primers to amplify *CSN4* gene for the construction of Flag-Csn4 vector |
| Flag-Csn4-R | CGCAGAGGAGCCTGAATGTTGAGTGGAATGATGTTATACAACAAGGTTTGCGGC |  |
| Flag-Csn6-F | CATGATATCGATTACAAGGATGACGATGACAAGATGACTACCACAACCACG | PCR primers to amplify *CSN6* gene for the construction of Flag-Csn6 vector |
| Flag-Csn6-R | CGCAGAGGAGCCTGAATGTTGAGTGGAATGATGTTCTTTGTAATGTTTTTTGC |  |
| Flag-Csn7-F | CATGATATCGATTACAAGGATGACGATGACAAGATGGAACAGACAAAGGC | PCR primers to amplify *CSN7* gene for the construction of Flag-Csn7 vector |
| Flag-Csn7-R | CGCAGAGGAGCCTGAATGTTGAGTGGAATGATGTCACAGCTTGCGCTTGCTCG |  |
| Flag-Csn1-ID-F | CTCGAAGAGTCAGTCTCCCTT | PCR primers for the identification of the in-frame Flag-Csn1 vector |
| Flag-Csn1-ID-R | CGTTGCCCATCTAGATTTGTG |  |
| Flag-Csn2-ID-F | CTCGAAGAGTCAGTCTCCCTT | PCR primers for the identification of the in-frame Flag-Csn2 vector |
| Flag-Csn2-ID-R | GACGGCCGTGTACTCCTTTC |  |
| Flag-Csn3-ID-F | CTCGAAGAGTCAGTCTCCCTT | PCR primers for the identification of the in-frame Csn3-Flag vector |
| Flag-Csn3-ID-R | GCAATAATGCACTGGCTACAGC |  |
| Flag-Csn4-ID-F | CTCGAAGAGTCAGTCTCCCTT | PCR primers for the identification of the in-frame Flag-Csn4 vector |
| Flag-Csn4-ID-R | GCGGTAGAGTCGTCTACTTC |  |
| Flag-Csn6-ID-F | CTCGAAGAGTCAGTCTCCCTT | PCR primers for the identification of the in-frame Flag-Csn6 vector |
| Flag-Csn6-ID-R | GAGTGATCAAGGACCTGCTC |  |
| Flag-Csn7-ID-F | CTCGAAGAGTCAGTCTCCCTT | PCR primers for the identification of the in-frame Flag-Csn7 vector |
| Flag-Csn7-ID-R | CTTGATACTTGCACGGTAGC |  |
| Actin-F | ATCCACGTCACCACTTTCAA | PCR primers to amplify the *ACTIN* gene in quantitative real-time PCR assays |
| Actin-R | TGCTTGGAGATCCACATTTG |  |
| Tri1-RT-F | CAGGAGAGTTATTGCCGAAG | PCR primers to amplify the *TRI1* gene in quantitative real-time PCR assays |
| Tri1-RT-R | ACTTTTCAGGGTTCTCGTAG |  |
| Tri5-RT-F | AGCAGATGGTTGCTGTCTTCT | PCR primers to amplify the *TRI5* gene in quantitative real-time PCR assays |
| Tri5-RT-R | TTCTGAGCCTCCTTCACATCG |  |
| Tri6-RT-F | AAATGCCCATTCCCTAGTTG | PCR primers to amplify the *TRI6* gene in quantitative real-time PCR assays |
| Tri6-RT-R | ATCTCGCATGTTATCCACCCT |  |
