# Supplementary material for: The COP9 signalosome complex regulates fungal development and virulence in the wheat scab fungus Fusarium graminearum
Source: Front Microbiol. 2023 Apr 24;14:1179676. doi: 10.3389/fmicb.2023.1179676 (PMC10165099; doi:10.3389/fmicb.2023.1179676)
Supplement: Supplementary file 2 [file Table_2.DOCX]

**Table S2. Identification of putative Csn complex in *F. graminearum***

| *Fusarium graminearum* | | | ***Aspergillus nidulans*** | |
| --- | --- | --- | --- | --- |
| Gene ID | Protein name in this study | Length of amino acid (aa) | Homology  in *A. nidulans* | Identity (%) |
| FGRAMPH1_01G06077 | Csn1 | 436 | Csn1 | 45.43 |
| FGRAMPH1_01G00499 | Csn2 | 491 | Csn2 | 72.32 |
| FGRAMPH1_01G07453 | Csn3 | 490 | Csn3 | 27.05 |
| FGRAMPH1_01G01579 | Csn4 | 420 | Csn4 | 48.54 |
| FGRAMPH1_01G06201 | Csn5 | 340 | Csn5 | 60.90 |
| FGRAMPH1_01G26709 | Csn6 | 419 | Csn6 | 42.93 |
| FGRAMPH1_01G03679 | Csn7 | 277 | Csn7 | 40.38 |
| *NA* | *-* | - | Csn8 | - |

*NA= Not avaliable*
