# Supplementary figures and images for: The COP9 signalosome complex regulates fungal development and virulence in the wheat scab fungus Fusarium graminearum
Source: Front Microbiol. 2023 Apr 24;14:1179676. doi: 10.3389/fmicb.2023.1179676 (PMC10165099; doi:10.3389/fmicb.2023.1179676)

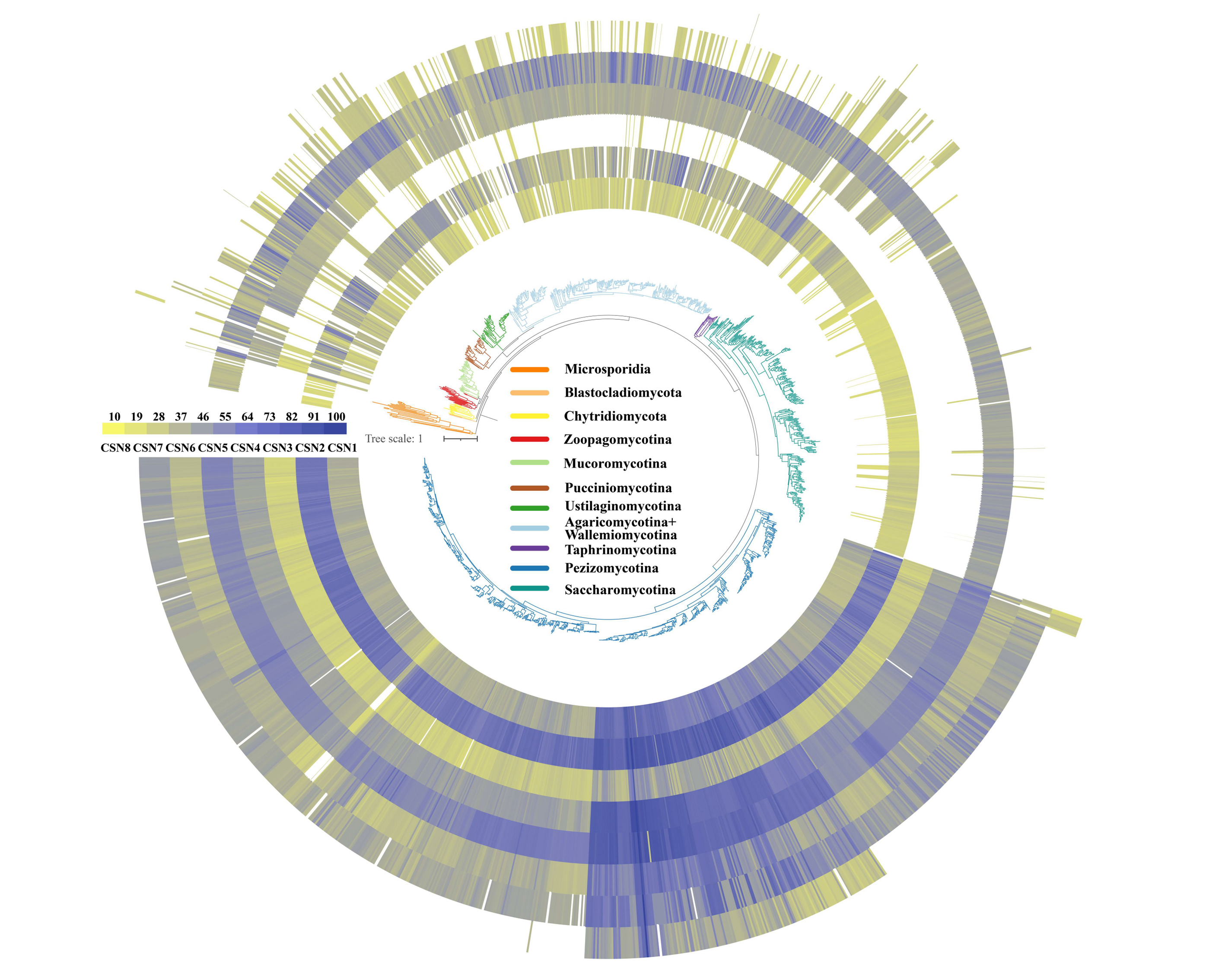

Supplement: Supplementary Figure 1 — Evolutionary conservation of the Csn subunits in fungi. [file Image_1.TIF]

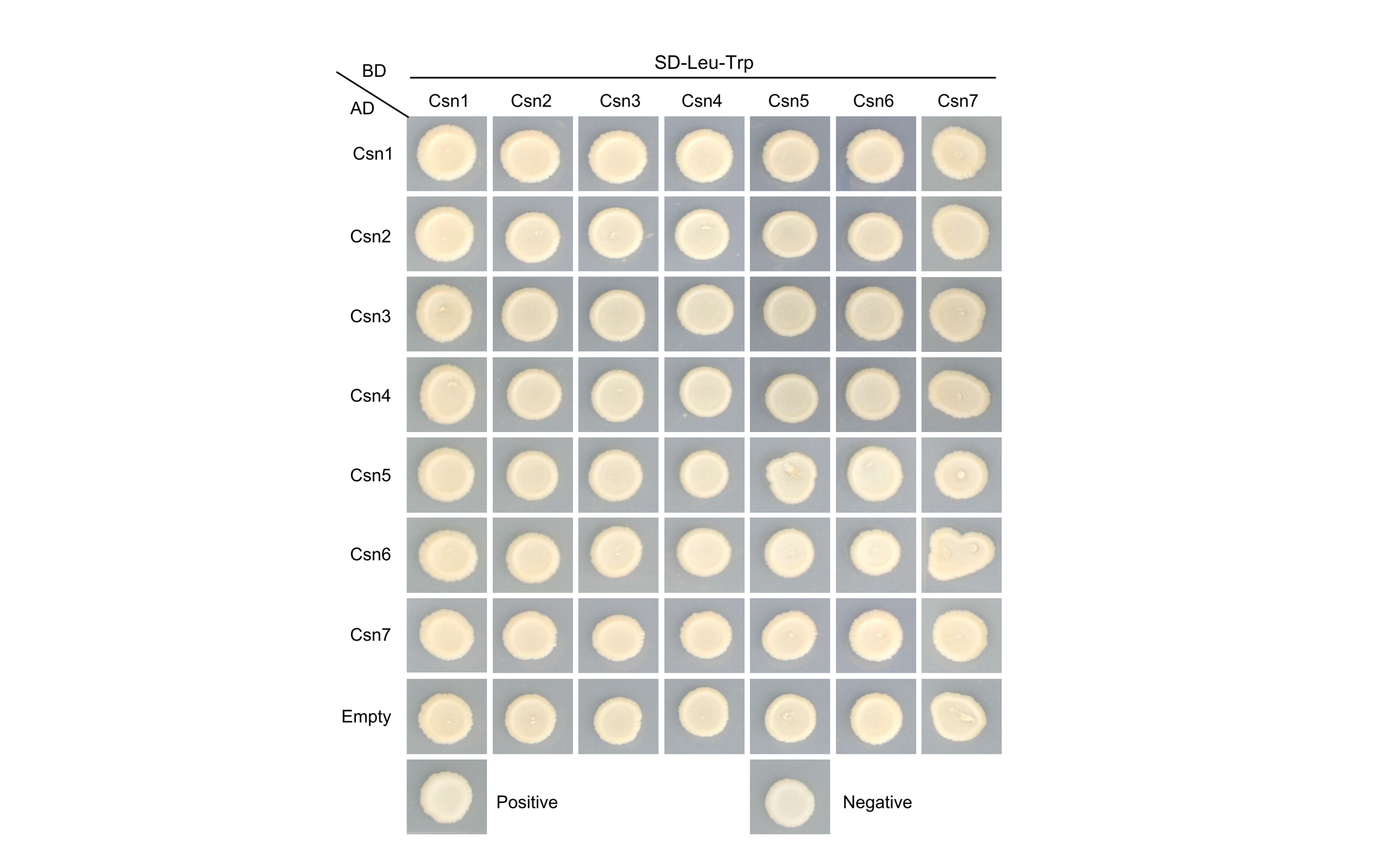

Supplement: Supplementary Figure 2 — Yeast transformants carrying the indicated constructs were plated onto selective plates supplemented without Leu/Trp to assay growth. [file Image_2.TIF]

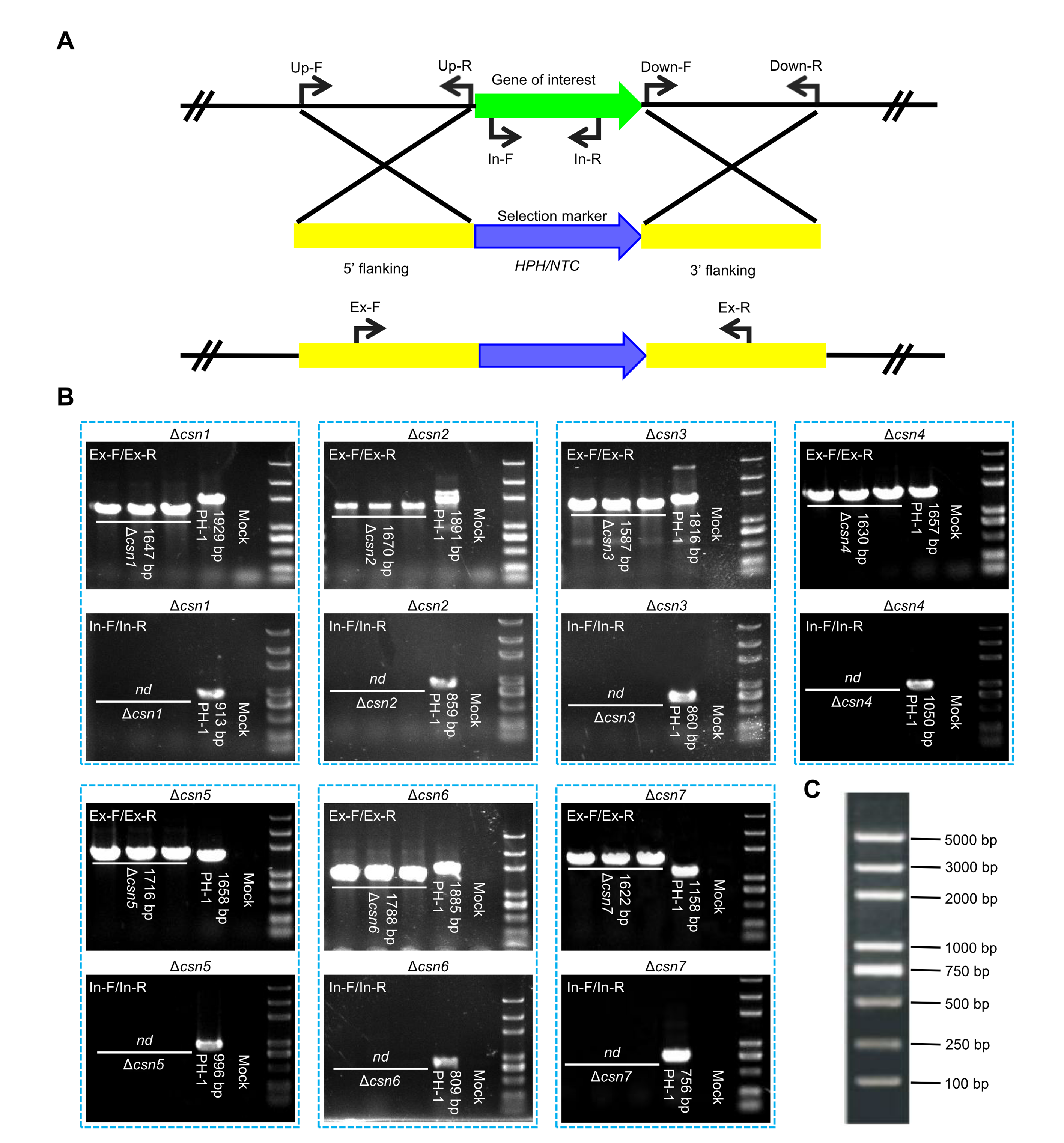

Supplement: Supplementary Figure 3 — Targeted gene deletion of the Csn complex in F. graminearum. (A) The Csn subunits and HPH genes are marked with large green and blue arrows, respectively. PCR primers are marked with small arrows. (B) Deletion mutants were identified by PCR assays. The size of the PCR products is indicated. (C) The DNA marker used in S3 (B). [file Image_3.TIF]

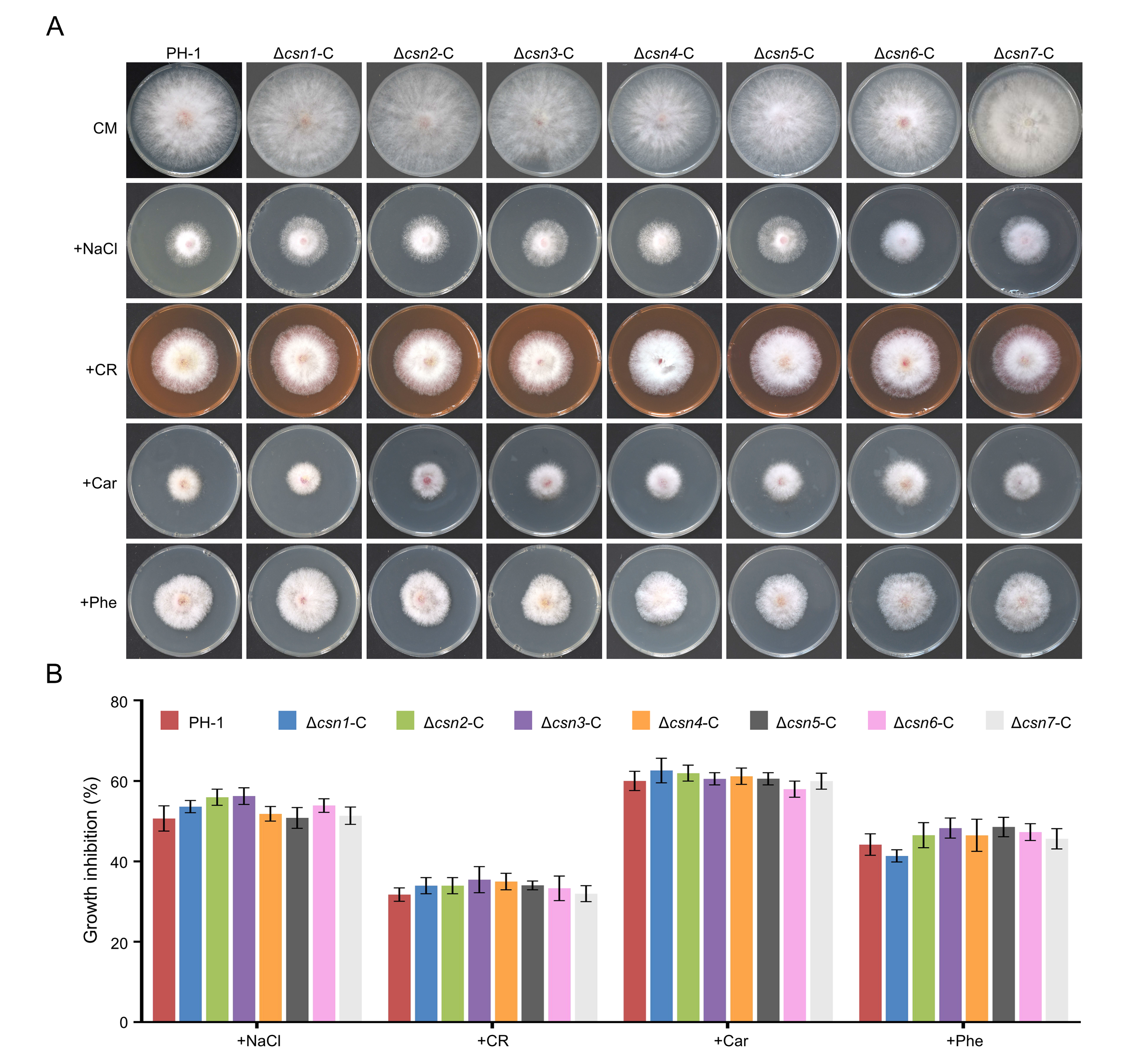

Supplement: Supplementary Figure 4 — The defect in the stress response was fully recovered to the wild-type level in the complementation strains of Csn mutants. (A) Morphologies of the indicated strains after incubation on complete medium (CM) supplemented with multiple abiotic stresses including NaCl, Congo red (CR), carbendazim (Car), and phenamacril (Phe). (B) The inhibition of the mycelial growth rate was examined after each strain was incubated for 3 days on complete medium supplemented with 1 M NaCl (B), 0.4 g/L Congo red (C), 0.5 μg/mL carbendazim (D) and 0.25 μg/mL phenamacril (E). Error bars indicate the standard deviation from three independent experiments. [file Image_4.TIF]
